# Knockdown of nicotinamide *N*-methyltransferase suppresses proliferation, migration, and chemoresistance of Merkel cell carcinoma cells in vitro

**DOI:** 10.1007/s13577-024-01047-0

**Published:** 2024-03-19

**Authors:** Valentina Pozzi, Elisa Molinelli, Roberto Campagna, Emma N. Serritelli, Monia Cecati, Edoardo De Simoni, Davide Sartini, Gaia Goteri, Nathaniel I. Martin, Matthijs J. van Haren, Eleonora Salvolini, Oriana Simonetti, Annamaria Offidani, Monica Emanuelli

**Affiliations:** 1 Department of Clinical Sciences, Polytechnic University of Marche, 60020 Ancona, Italy; 2 Department of Clinical and Molecular Sciences, Polytechnic University of Marche, 60020 Ancona, Italy; 3 Department of Biomedical Sciences and Public Health, Polytechnic University of Marche, 60020 Ancona, Italy; 4 Biological Chemistry Group, Institute of Biology Leiden, Leiden University, Sylviusweg 72, 2333 BE Leiden, The Netherlands; 5 New York-Marche Structural Biology Center (NY-MaSBiC), Polytechnic University of Marche, 60131 Ancona, Italy

**Keywords:** Nicotinamide *N*-methyltransferase, Merkel cell carcinoma, Gene silencing, Proliferation and migration, Chemosensitivity, Molecular biomarker

## Abstract

Merkel cell carcinoma (MCC) is an aggressive skin cancer, with a propensity for early metastasis. Therefore, early diagnosis and the identification of novel targets become fundamental. The enzyme nicotinamide *N*-methyltransferase (NNMT) catalyzes the reaction of *N*-methylation of nicotinamide and other analogous compounds. Although NNMT overexpression was reported in many malignancies, the significance of its dysregulation in cancer cell phenotype was partly clarified. Several works demonstrated that NNMT promotes cancer cell proliferation, migration, and chemoresistance. In this study, we investigated the possible involvement of this enzyme in MCC. Preliminary immunohistochemical analyses were performed to evaluate NNMT expression in MCC tissue specimens. To explore the enzyme function in tumor cell metabolism, MCC cell lines have been transfected with plasmids encoding for short hairpin RNAs (shRNAs) targeting NNMT mRNA. Preliminary immunohistochemical analyses showed elevated NNMT expression in MCC tissue specimens. The effect of enzyme downregulation on cell proliferation, migration, and chemosensitivity was then evaluated through MTT, trypan blue, and wound healing assays. Data obtained clearly demonstrated that NNMT knockdown is associated with a decrease of cell proliferation, viability, and migration, as well as with enhanced sensitivity to treatment with chemotherapeutic drugs. Taken together, these results suggest that NNMT could represent an interesting MCC biomarker and a promising target for targeted anti-cancer therapy.

## Introduction

Merkel cell carcinoma (MCC) is a rare neuroendocrine tumor belonging to the non-melanoma skin cancers. Incidence rates of MCC range from 0.1 to 2.5 cases per 100,000 individuals although in the last decades, in different countries, an increase incidence per year has been reported. This tumor usually arises as a fast-growing pink–red dome-shaped nodule, mostly occurring on sun-exposed areas [1]. The name MCC has been historically used due to the structural and morphological characteristics that these tumors cells share with Merkel cells, although recent studies suggested different origins of MCC cells [2].

MCC most commonly develops on the sun-exposed skin of the head and neck (50%), in upper limbs or shoulders (24%), lower limb or hips (15%), trunk (11%) and more rarely in other districts. Risk factors can be viral infection, DNA damage and mutation, as the result of ultraviolet exposure, old age, male sex, and individuals with immunosuppression [2]. Almost 95% patients are fair skinned and MCC is known to be rare in dark-skin individuals. MCC of the head and neck district is very aggressive since it is characterized by a high frequency of recurrence and it is prone to develop early metastases. Indeed, MCC displays a significantly higher 5-year recurrence rate compared to other aggressive skin cancers like melanoma or squamous cell carcinoma [3].

The initial approach for the management of MCC depends on the pathology of the primary cancer and the presence of metastasis. Surgical excision is the primary treatment, with wider margin excisions in case of presence of baseline risk factors. Non-surgical treatment of MCC includes radiotherapy, immunotherapy, and chemotherapy. MCC displays a marked radiosensitivity, thus radiotherapy is effective in controlling the malignancy though systemic relapses are frequent, making the radiotherapy recommended only for patients that are not surgical candidates. Recently, the use of checkpoint immunotherapy has demonstrated to be beneficial for MCC patients, although more finalized to control the disease than reaching a curative aim [4]. MCC is recognized as a chemosensitive malignancy displaying high response rates; thus, chemotherapy is used for the primary therapy of advanced MCC or as an adjuvant treatment. Nonetheless, the duration of response to chemotherapy is poor, with patients suffering from significant toxicity to the therapy [5]. Therefore, it is of outmost importance the identification of new diagnostic and prognostic markers, as well as biomolecules that could be used as target for precision medicine.

In this work, we focused on nicotinamide *N*-methyltransferase (NNMT), a phase II metabolism enzyme which has found to be overexpressed in several malignancies, contributing to tumor progression [6–12].

The aim of the present work was to explore the potential involvement of this enzyme in MCC. Immunohistochemistry was performed on a few selected formalin-fixed and paraffin-embedded (FFPE) MCC tissue samples, to evaluate NNMT expression levels. To further investigate the role played by the enzyme in tumor cell metabolism, MCC-13 and MCC-26 Merkel carcinoma cell lines have been transfected with vectors encoding short hairpin RNAs targeting NNMT mRNA, and the efficiency of enzyme knockdown has been assessed by real-time PCR and western blot analysis. Subsequently, we analyzed the impact of NNMT downregulation on MCC cell lines in terms of proliferation, cell viability, and invasiveness. Finally, cell viability of NNMT downregulating cells and controls was analyzed upon treatment with cisplatin, to evaluate the potential involvement of the enzyme in sensitivity of MCC cells to chemotherapy.

## Materials and methods

### Immunohistochemistry

A retrospective analysis was conducted in accordance with the principles of the Declaration of Helsinki on a total of 11 FFPE MCC specimens, collected from patients undergoing biopsy or excisional surgery between 2018 and 2020. Tissue samples were obtained from the archives of the Section of Pathology, Department of Biomedical Sciences and Public Health, Polytechnic University of Marche.

5 µm sections from FFPE blocks were processed as previously described [13]. Analyses were independently performed by two investigators blinded to the patient group (E.S. and G.G.), using a Nikon Eclipse E600 light microscope equipped with a Nikon DS-Vi1 digital camera (Nikon Instruments, Europe BV, Kingston, Surrey, England). Stained cells were counted in at least ten fields per sample (field area: 0.07 mm^2^, magnification: × 400) and quantified as a percentage of the total counted cells. Agreement between observers was always > 95%. Discrepancies were resolved by simultaneous reexamination of the slides, using a double-headed microscope.

### Cell lines and culture conditions

MCC-13 [14] and MCC-26 [15] cells were cultured in Dulbecco’s modified Eagle medium (DMEM) High Glucose media added with 10% fetal bovine serum (FBS) (Euroclone, Milan, Italy) and 50 μg/mL gentamicin, at 37℃ in a humified 5% CO_2_ incubator.

### NNMT shRNA-mediated gene silencing

To achieve NNMT knockdown, MCC-13 and MCC-26 cells were seeded in 24-well plates (7 × 10^4^ cells/well) and grown in complete medium until approximately 80% confluence at the moment of transfection, that was performed using FuGENE HD Transfection Reagent (Promega, Madison, WI, USA). The day after, cells were transfected with vectors (0.5 µg/well) encoding short hairpin RNAs (shRNAs) targeting different regions of NNMT transcript (pLKO.1–164, target nucleotide sequence 5ʹ-ACCCTCGGGATTACCTAGAAA-3ʹ, pLKO.1–330, target nucleotide sequence 5ʹ-CCTCTCTGCTTGTGAATCCTT-3ʹ, and pLKO.1–448, target nucleotide sequence 5ʹ- GTGACCTATGTGTGTGATCTT-3ʹ). Control cells were transfected with empty vector (pLKO.1-puro) or treated with transfection reagent only (mock). 48 h following transfection, cellular clones stably downregulating NNMT started to be selected in complete medium containing 1 μg/mL puromycin, to select, for each sample, those cellular clones containing vector conferring resistance to puromycin. For this reason, medium was changed every 2 days, until the complete death of mock cells, while puromycin-resistant cells were maintained in selection medium. NNMT knockdown efficiency was analyzed by real-time PCR and western blot analysis.

### Real-time quantitative PCR

RNA was extracted from cancer cell pellets (1 × 10^6^) using the SV Total RNA Isolation System (Promega, Madison, WI, USA). 2 μg of RNA was reverse transcribed with M-MLV Reverse Transcriptase (Promega, Madison, WI, USA) and 1 µL of the cDNA mixture was used for real-time PCR, as previously described [16]. Fold changes in relative gene expression were calculated by 2^−ΔΔCt^, where ΔCt = Ct (NNMT) − Ct (housekeeping gene) and Δ(ΔCt) = ΔCt (NNMT silencing vector) − ΔCt (empty vector).

### Western blot analysis

Western blot analysis was set up to evaluate NNMT protein levels, as reported elsewhere [16]. Blot was probed with rabbit polyclonal antibody against NNMT (Sigma-Aldrich; cat. No. SAB1100302-200UL) (1:1,000 dilution) or with mouse monoclonal antibody against glyceraldehyde-3-phosphate dehydrogenase (GAPDH) (Santa Cruz Biotechnology; cat. No. sc-47724) (1:250), for 1 h, followed by incubation with horseradish peroxidase (HRP)-conjugated goat anti-rabbit IgG (Sigma-Aldrich; cat. No. A0545-1ML) (1:150,000 dilution) or with HRP-conjugated goat anti-mouse IgG (Bio-rad; cat. No. 1706516) (1:5000) for 1 h at room temperature, respectively.

### Cell viability and proliferation assays

The assay based on 3-(4,5-dimethylthiazol-2-yl)-2,5-diphenyl tetrazolium bromide (MTT) was used to evaluate cell viability, measuring the capability of mitochondrial dehydrogenase enzymes of living cells to convert tetrazolium salt MTT to formazan. The MTT test was executed in accordance with a previous study [16]. Proliferation of NNMT-silenced cells was estimated by trypan blue exclusion assay. MCC-13 and MCC-26 cells were seeded on 6-well plates at a density of 1 × 10^5^ cells/well in serum-free medium. The day after, representing the 0 h time point, cells were detached using 500 μL trypsin and centrifuged at 300×*g* for 3 min. Cell pellet was resuspended in 500 μL complete medium, trypan blue was added, and cells were counted using Burker’s chamber. The same procedure was repeated at different time points (24, 48, and 72 h). The number of viable cells was indicated as percentage of the control (number of viable counted cells of each sample at 0 h, corresponding to 100%) and presented as mean values ± standard deviation. Each experiment, in triplicate, was repeated 3 times.

### Monolayer wound healing assay

To evaluate cell migration ability, transfected cells were seeded into 6-well plates (3 × 10^5^ cells/well) until 100% confluency. A 200 μL sterile pipette tip was used to obtain the wound on cells monolayers, then cell debris were removed by washing cell monolayers 3 times and the medium was replaced with DMEM High Glucose media containing 0.5% FBS. Monolayers were photographed at 0, 2, 4, 8, 24 h after scratching. Each experiment, in triplicate, was repeated 3 times.

### Chemotherapeutic treatment

Concerning the evaluation of the effect induced by NNMT silencing on cell sensitivity to chemotherapy, MCC-26 cells, both those downregulating the enzyme and controls, were treated with cisplatin at different concentrations (0.1 and 1 μg/mL), and the sensitivity to drug was evaluated through MTT assay in accordance with what was previously described [16]. Briefly, cells were seeded in 96-well plates (5 × 10^3^ cells/well). The day after seeding, MCC-26 cells were treated with cisplatin. MTT assay was used to estimate the cell proliferation at different time points (24, 48, and 72 h) after starting treatment with drug.

### Statistical analysis

GraphPad Prism software version 8.00 for Windows (GraphPad Software, San Diego, CA, USA) was used for statistical analysis. Kruskal–Wallis test was used to evaluate differences between groups. A *p* value < 0.05 was considered as statistically significant.

## Results

### NNMT expression in MCC tissue samples

**Fig. 1:**
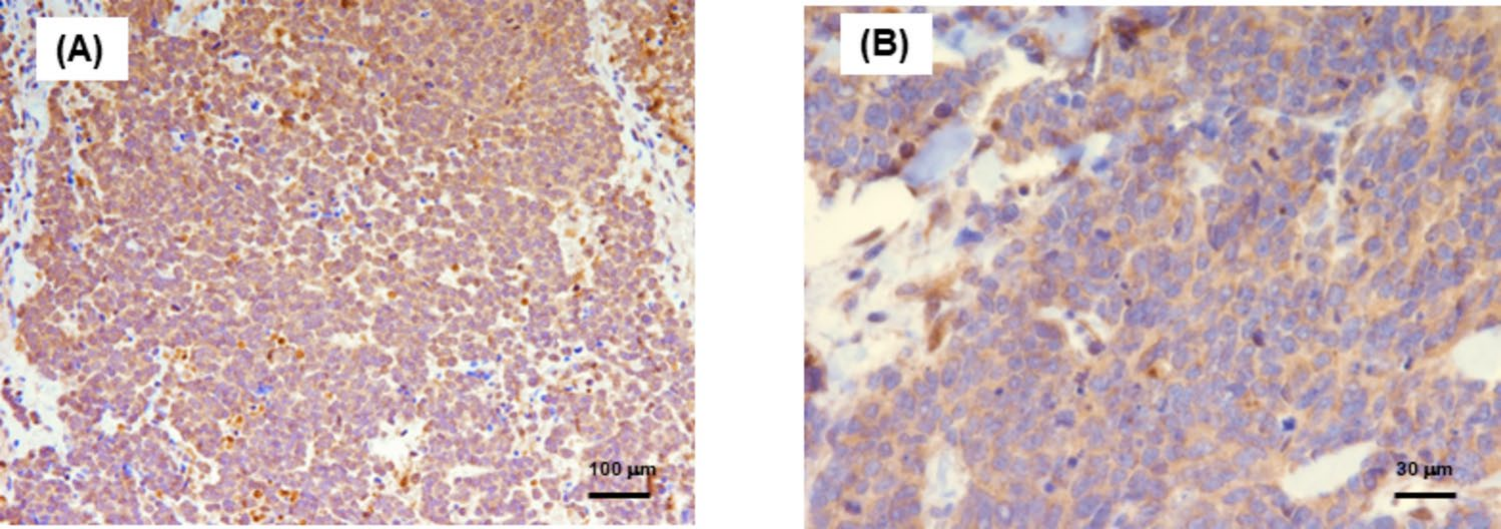
Low (A: scale bar 100 μm) and high (B: scale bar 30 μm) magnification images showing NNMT immunohistochemical expression in human Merkel cell carcinoma sections

The results obtained by immunohistochemical evaluation evidenced a strong NNMT cytoplasmic expression in more than 90% of the tumor cells in the analyzed fields, while the nuclei showed no immunostaining (Fig. 1).

### Efficiency of NNMT silencing in MCC cell lines

**Fig. 2:**
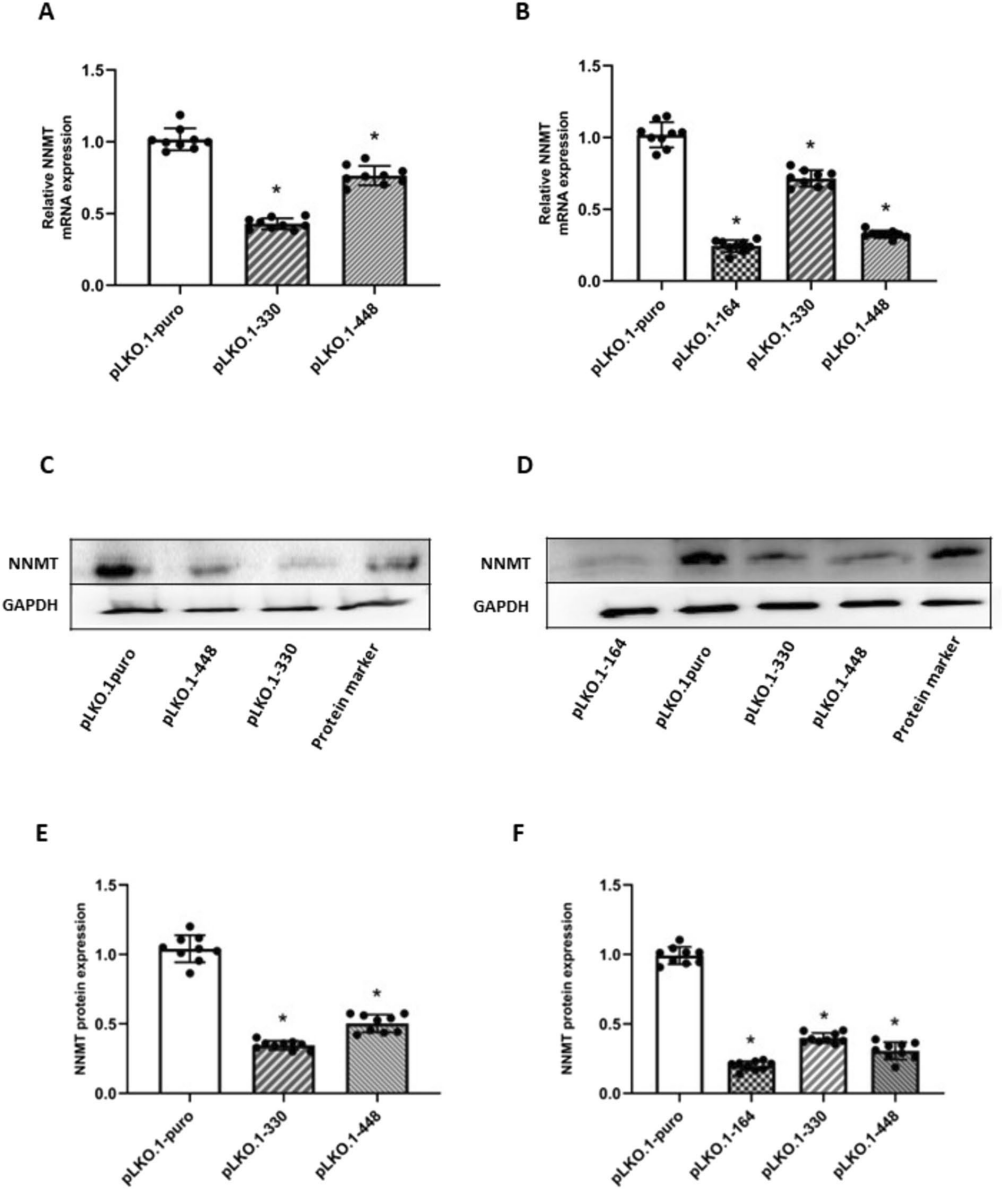
Evaluation of NNMT silencing in MCC cell lines. MCC-13 (A, C, E) and MCC-26 (B, D, F) cells were transfected with plasmids coding shRNAs targeting NNMT (pLKO.1–164, pLKO.1–330 and pLKO.1–448) and with empty vector as control (pLKO.1-puro). Enzyme expression was then evaluated at mRNA and protein level, by real-time PCR (A, B) and western blot (C, D), followed by densitometry (E, F). Human recombinant his-tagged NNMT and human recombinant his-tagged GAPDH were used as protein standards (29.6 kDa and 36 kDa molecular mass, respectively). Each experiment, in triplicate, was repeated 3 times. Values are expressed as mean ± standard deviation (*p < 0.05)

Real-time PCR analyses showed that NNMT-silenced MCC-13 cells showed a significant (*p* < 0.05) enzyme downregulation (0.43 ± 0.039 for pLKO.1–330 and 0.75 ± 0.067 for pLKO.1–448) compared to control cells (1.00 ± 0.078 pLKO.1-puro) (Fig. 2A). In MCC-26 cell line, treatment with pLKO.1–164 (0.24 ± 0.036), pLKO.1–330 (0.69 ± 0.052), and pLKO.1–448 (0.31 ± 0.029) led to a significant reduction of enzyme expression with respect to reference sample (1.00 ± 0.085; pLKO.1-puro) (Fig. 2B). Western blot analysis and densitometry of immunoreactive bands confirmed a decreased NNMT expression in MCC-13 cells transfected with pLKO.1–330 (0.36 ± 0.038) and pLKO.1–448 (0.48 ± 0.058) compared with those treated with empty vector (1.00 ± 0.089; pLKO.1-puro) (Fig. 2C and E), as well as in MCC-26 cells transfected with pLKO.1–164 (0.21 ± 0.032), pLKO.1–330 (0.38 ± 0,042), and pLKO.1–448, (0.30 ± 0.047) with respect to control cells (1.00 ± 0.076; pLKO.1-puro) (Fig. 2D and F).

### Effect of NNMT downregulation on proliferation, viability, and migration of MCC cells

**Fig. 3:**
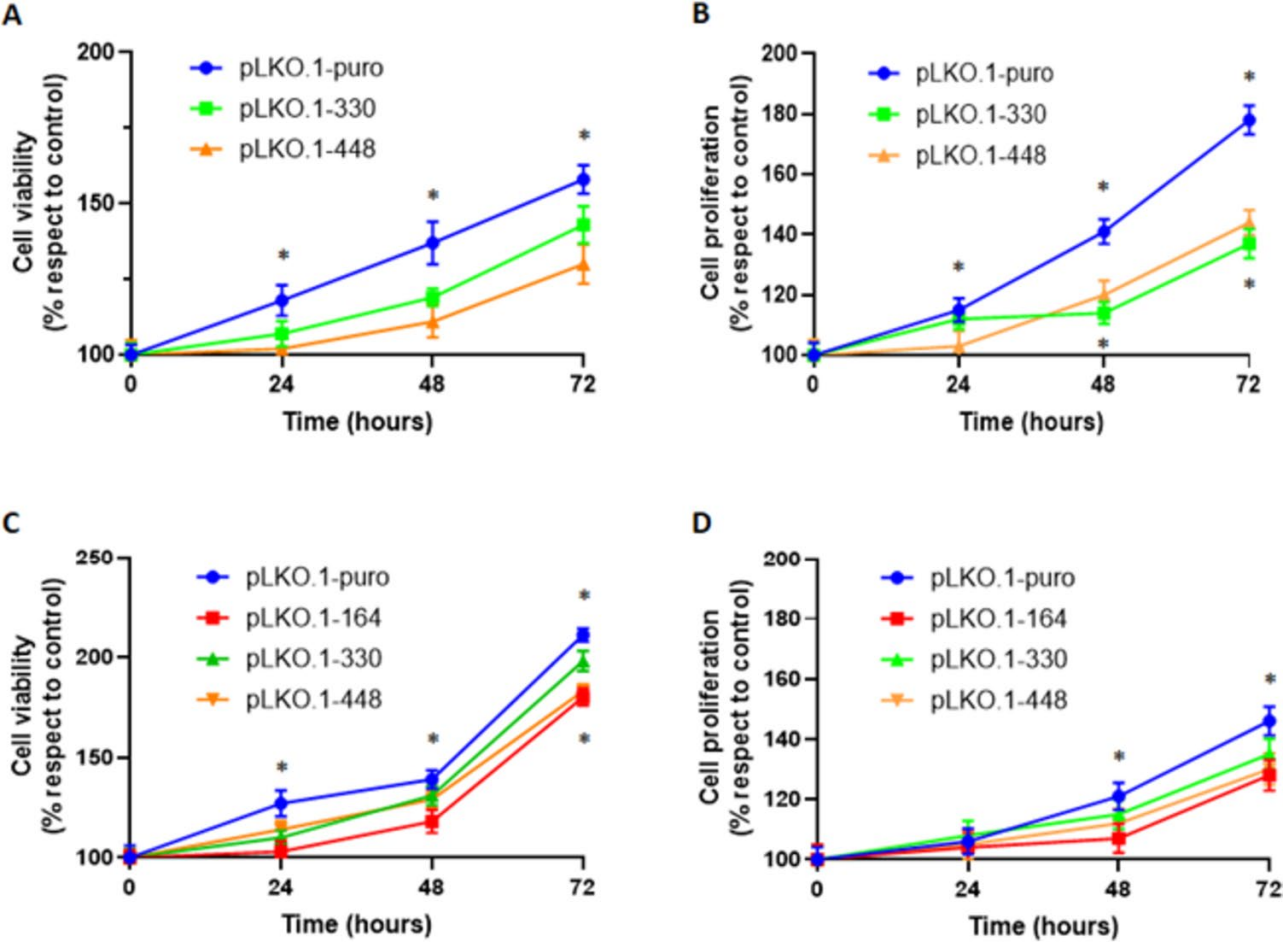
In vitro effect of NNMT silencing on cell viability and proliferation. Cell viability was measured by MTT (A, C) and trypan blue exclusion (B, D) assays, in both MCC-13 (A, B) and MCC-26 (C, D) cell lines. Each experiment, in triplicate, was repeated 3 times. Values are expressed as mean ± standard deviation (*p < 0.05)

In MCC-13 cells downregulating NNMT (pLKO.1–330 and pLKO.1–448), data obtained clearly showed a significant (*p* < 0.05) reduction in cell growth after 24, 48, and 72 h compared to control cells (pLKO.1-puro) (Fig. 3A). In MCC-26 cells, enzyme downregulation resulted in significant reduction of cell growth after 24, 48, and 72 h in cells treated with pLKO.1–164, and only after 72 h in cells treated with pLKO.1–330 and pLKO.1–448 (Fig. 3C). Results collected from the trypan blue exclusion assay revealed that NNMT silencing led to a significant (*p* < 0.05) decrease of cell proliferation in both MCC-13 (Fig. 3B) and MCC-26 (Fig. 3D) cells harboring shRNA plasmids against NNMT with respect to that of reference samples (pLKO.1-puro) at 48 and 72 h time points.

**Fig. 4:**
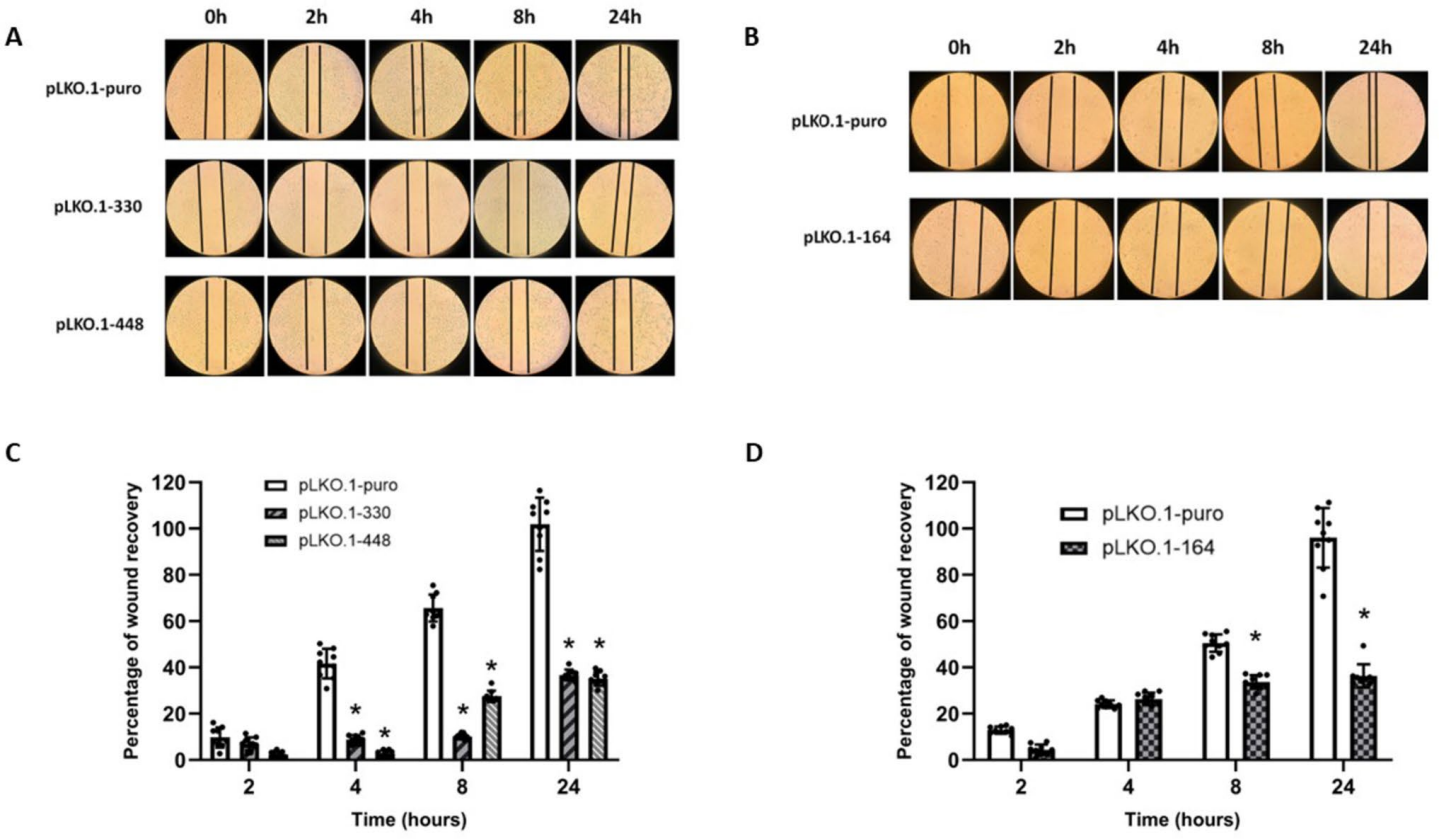
In vitro effect of NNMT silencing on cell migration. MCC cells were subjected to wound healing assay to evaluate their migration potential. MCC-13 cells transfected with plasmids pLKO.1–330, pLKO.1–448, or pLKO.1-puro (A) and MCC-26 cells transfected with plasmids pLKO.1–164, or pLKO.1-puro (B) were photographed immediately after scratch (0 h) and at different time points, ranging between 2 and 24 h. Migration ability was evaluated by measuring percentage of wound recovery compared with 0 h, as reported in bar diagrams (C or MCC-13 and D for MCC-26). Each experiment, in triplicate, was repeated 3 times. Values are expressed as mean ± standard deviation (*p < 0.05)

Migration capability of MCC-13 and MCC-26 cells was monitored by monolayer wound healing assay. For each sample, data were reported as percentage of wound recovery with respect to 0 h. Compared with control cells, the migration rate of both cells lines significantly (*p* < 0.05) decreased after NNMT silencing at 4, 8, and 24 h time points for MCC-13 cells (Fig. 4A and C) and at 8 and 24 h time points for MCC-26 cells (Fig. 4B and D).

### Effect of NNMT knockdown on MCC cells sensitivity to chemotherapeutic treatment

**Fig. 5:**
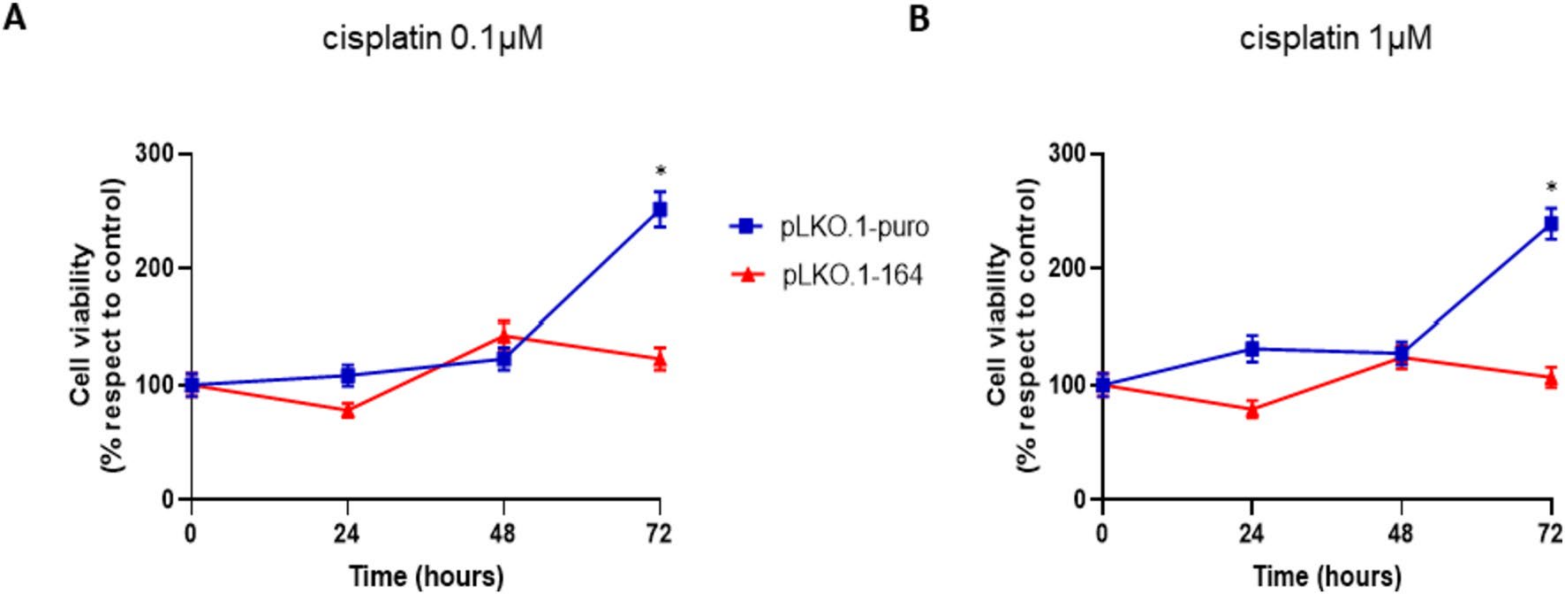
Impact of NNMT knockdown on chemosensitivity of MCC-26 cells. The effect of cisplatin treatment at 0.1 µg/mL (A) and 1 µg/mL (B) concentrations on viability of MCC-26 cells was evaluated by MTT assay. Measurements were performed at 0, 24, 48, and 72 h time points. Each experiment, in triplicate, was repeated 3 times. All values are expressed as mean ± standard deviation (*p < 0.05)

Several studies reported in literature clearly demonstrated an NNMT involvement in resistance to chemotherapy. Therefore, MCC-26 cells downregulating NNMT, as well as controls, were treated with cisplatin at different concentration values and then cell viability was assessed using MTT assay. Interestingly, upon treatment with cisplatin at 0.1 and 1 μM, a significantly (*p* < 0.05) higher decrease in cell viability was shown in NNMT downregulating cells compared with control cells at 72 h time point (Fig. 5).

## Discussion

MCC is a rare and extremely aggressive cutaneous malignancy, presenting the highest case-by-case mortality rate among all skin tumors [1]. The clinical presentation of MCC is varied and nonspecific, usually presenting as a rapidly growing solitary cutaneous or subcutaneous erythematous or violaceous nodule [17]. Besides rapid local growth, cancer progression is so fast, with metastasis to the lymph nodes and distal organs. Despite advances in the understanding of the carcinogenesis of MCC, the molecular mechanisms underlying this disease are far to be fully elucidated. Patients are often elderly and the metastatic potential of the tumor is high, so MCC prognosis is poor [18–20]. An early diagnosis should be a primary goal and the development of targeted therapies is urgently required.

In the present study, we focused on NNMT enzyme, catalyzing the N-methylation of nicotinamide, pyridines, and other analogs compounds, thus playing an important activity in the transformation of many xenobiotics [21, 22].

Preliminary immunohistochemical analyses, performed in a few selected FFPE MCC tissue samples, showed high NNMT expression. Subsequently, the influence of shRNA-mediated NNMT gene silencing on viability, migration, and proliferation of MCC cell lines was evaluated. Upon assessment of the efficiency of NNMT knockdown by real-time PCR and western blot assay, the effect on cell growth, viability, and migration was analyzed by MTT, trypan blue, and wound healing assays, respectively. Interestingly, our data showed that efficient downregulation of NNMT resulted in significantly reduced cell proliferation and viability, as well as migration and invasiveness. To speculate the relation between enzyme expression and resistance to chemotherapeutic drugs, cell viability of MCC cells downregulating NNMT and controls was evaluated under treatment with increasing concentrations of cisplatin. Interestingly, preliminary data showed that enzyme knockdown significantly enhanced decrease of cell viability following administration with chemotherapeutic drug, suggesting that targeting NNMT could represent a promising strategy to increase the efficacy of chemotherapy.

In our previous works, we reported an enhanced expression of NNMT in numerous cancers, such as renal cell carcinoma (ccRCC) [23], oral squamous cell carcinoma (OSCC) [11], bladder urothelial carcinoma [9], non-small cell lung cancer (NSCLC) [24], and skin malignancies [8, 25].

Although different kind of cancers have been correlated with NNMT dysregulation, its role in cancer cell metabolism remains partly undiscovered. As found in multiple types of human malignancies, NNMT seems to promote both tumorigenicity and proliferation of tumor cells. Many studies have been carried out to elucidate the function of the NNMT in tumorigenesis and cancer cell metabolism, as well as to clarify the significance of its overexpression in different malignancies [26–35].

In human bladder cancer cell lines, the enzyme silencing led to a significant decrease in cell migration, highlighting the crucial role of the NNMT in tumor metastasis and invasion [30]. Analyses carried out on ccRCC cells treated with shRNAs against NNMT mRNA showed that the enzyme can promote cellular invasiveness both in vitro and in vivo, with the activation of the PI3K/Akt/SP1/MMP-2 pathway [31]. Furthermore, NNMT knockdown in the pancreatic cancer cell line PANC-1 was significantly correlated with reduction of cell proliferation and migration, as well as invasive capacity, suggesting the enzyme involvement in cell proliferation and metastatic potential of tumor cells [32]. In vitro and in vivo experiments of NNMT silencing in Bcap-37 and MDA-MB-231 human breast cancer cell lines showing high enzyme levels led to apoptosis induction and significantly reduced cell growth and tumorigenicity. Converse results were obtained after NNMT upregulation in MCF-7 and SK-BR-3 breast cancer cell lines that lacked constitutive NNMT expression [27]. Equally, NNMT upregulation had been induced in SW480 colorectal cancer cells lines which lack endogenous NNMT expression, whereas NNMT silencing has been performed in HT-29 cells with high constitutive enzyme expression, showing that NNMT enhances cell proliferation and colony formation, inhibits apoptotic pathway, promotes cell cycle, and increases ATP levels [28]. In NSCLC H1993 cells, which displayed high endogenous levels of the enzyme, NNMT knockdown significantly suppressed colony formation capability [36]. In line with these findings, in the PC3 prostate cancer cells, induction of NNMT overexpression promoted cell proliferation, invasive ability, and migration capability, by increasing mRNA levels of histone deacetylase SIRT1 ability [37]. Similarly, treatment of EC9706 and TE1 esophageal squamous cell carcinoma cells with shRNAs targeted to NNMT mRNA significantly suppressed cell migration and viability, promoted apoptosis and cell cycle, inhibited EMT via the Wnt/β-catenin pathway [38]. Our previous studies demonstrated that NNMT silencing in human OSCC and NSCLC cell lines significantly inhibited cell growth and tumorigenicity [39, 40]. Conversely, the induction of NNMT overexpression in the HSC-2 OSCC cell line was significantly associated with enhanced cell proliferation [41], suggesting that the enzyme could play a fundamental role in the tumorigenic capability and proliferation of tumor cells, and the possibility that NNMT could represent a therapeutic target for the management of several solid malignancies.

Currently, the specific mechanism by which NNMT can influence tumorigenesis and tumor progression has not been completely clarified. NNMT could modify the SAM:SAH ratio affecting the methylation state of cancer cells, but also store N1-methylnicotinamide into tumor cells, thus fine-tuning the methylation state of cancer cells [42]. Moreover, NNMT activity is involved in the regulation of the homeostasis of nicotinamide, a specific precursor of the NAD + . Therefore, the catalytic activity of the NNMT can control the amount of nicotinamide inside the cell available for the energy metabolism, thus influencing and modulating multiple pathways linked to both death and viability [43]. These pathways involve NAD-dependent PARPs and SIRT1 enzymes, that are important for the correct DNA repair which prevents the tumor transformation [43, 44].

Different studies reported in literature clearly demonstrated an NNMT involvement in resistance to chemotherapy. Enzyme downregulation decreased resistance to 5-fluorouracil (5-FU) in human colorectal cell line HT-29, while NNMT overexpression induced the opposite effect in the colorectal cell line SW480. The capability of the enzyme to increase chemosensitivity was found to be mediated by its reaction product N1-methylnicotinamide, which reduces the production of reactive oxygen species induced by 5-FU treatment [28]. Furthermore, a recent work showed that NNMT knockdown increases 5-FU sensitivity of esophageal squamous cell carcinoma cell line TE1 via suppressing Warburg effect, while overexpression of the enzyme in EC1 and Eca109 cells increased the 5-FU sensitivity, thus suggesting that NNMT can represent a potential therapeutic target to enhance the therapeutic activity of 5-FU [45]. Results obtained in our recent study clearly demonstrated that NNMT knockdown led to a significant reduction of cell proliferation and migration of melanoma cell lines. Moreover, the enzyme downregulation in melanoma cells was associated with an increased sensitivity to treatment with dacarbazine, suggesting that NNMT could be involved in mechanisms promoting melanoma cell resistance to chemotherapy [16].

The present study is in line with the available literature ascribing to the NNMT, an important role in promoting cancer cell proliferation, invasiveness, and chemoresistance. Nonetheless, we are aware of the limitations of our study. The immunohistochemical analyses performed in this study involved a limited cohort of patients since MCC is an extremely rare malignancy; thus, further investigations should be focused on evaluating the immunohistochemical expression of NNMT in a larger cohort of patients. Moreover, it would be necessary to validate our interesting in vitro findings in an in vivo model, to confirm the possibility of exploiting a targeted anti-cancer therapy based on the use of specific inhibitors of the enzyme in the management of MCC. In this context, studies providing a mechanistic insight would also be necessary.

## Conclusion

This is the first study that highlights the involvement of NNMT in MCC, supporting the hypothesis that the enzyme could represent an interesting biomarker for treatment and detection of this kind of tumor. Indeed, based on the results obtained from cellular experiments, NNMT seems to be involved in tumor cell proliferation, migration, and resistance to chemotherapy, thus representing a potential target for counteracting tumor growth. Although further experiments are necessary to understand the specific mechanisms by which NNMT could participate to MCC tumorigenesis, our results seem to indicate that this enzyme may provide an interesting molecular target for cancer therapy.

## Data Availability

The data that support the findings of this study are available from the corresponding authors, DS and RC, upon reasonable request.

## Abbreviations

ccRCC: Clear cell renal cell carcinoma
DMEM: Dulbecco’s modified Eagle medium
FBS: Fetal bovine serum
FFPE: Formalin-fixed and paraffin-embedded
GAPDH: Glyceraldehyde-3-phosphate dehydrogenase
HRP: Horseradish peroxidase
MCC: Merkel cell carcinoma
MTT: 3-(4,5-Dimethylthiazol-2-yl)-2,5-diphenyl tetrazolium bromide
NNMT: Nicotinamide *N*-methyltransferase
NSCLC: Non-small cell lung cancer
OSCC: Oral squamous cell carcinoma
shRNAs: Short hairpin RNAs
